# Widespread diminishing anthropogenic effects on calcium in freshwaters

**DOI:** 10.1038/s41598-019-46838-w

**Published:** 2019-07-18

**Authors:** Gesa A. Weyhenmeyer, Jens Hartmann, Dag O. Hessen, Jiří Kopáček, Josef Hejzlar, Stéphan Jacquet, Stephen K. Hamilton, Piet Verburg, Taylor H. Leach, Martin Schmid, Giovanna Flaim, Tiina Nõges, Peeter Nõges, Valerie C. Wentzky, Michela Rogora, James A. Rusak, Sarian Kosten, Andrew M. Paterson, Katrin Teubner, Scott N. Higgins, Gregory Lawrence, Külli Kangur, Ilga Kokorite, Leonardo Cerasino, Clara Funk, Rebecca Harvey, Florentina Moatar, Heleen A. de Wit, Thomas Zechmeister

**Affiliations:** 1 Department of Ecology and Genetics/Limnology, Uppsala University, Norbyvägen 18D, 752 36 Uppsala, Sweden; 2 Institute for Geology, Center for Earth System Research and Sustainability (CEN), University of Hamburg, Bundesstraße 55, 20146 Hamburg, Germany; 3 Department of Biosciences, Centre for Biogeochemistry in the Anthropocene (CBA), University of Oslo, Box 1066, Blindern, 0316 Norway; 4 Institute of Hydrobiology, Biology Centre CAS, Na Sádkách 7, 370 05 České Budějovice, Czech Republic; 5 INRA CARRTEL, 75 bis avenue de Corzent, 74203 Thonon-les-Bains, cx France; 6 Kellogg Biological Station and Department of Integrative Biology, Michigan State University, Hickory Corners, MI 49060 and Cary Institute of Ecosystem Studies, Millbrook, NY 12545 USA; 7 National Institute of Water and Atmospheric Research, Hamilton, New Zealand; 8 Department of Biological Sciences, Rensselaer Polytechnic Institute, Troy, NY 12180 USA; 9 Surface Waters – Research and Management, Eawag: Swiss Federal Institute of Aquatic Science and Technology, Seestrasse 79, 6047 Kastanienbaum, Switzerland; 10 Department of Sustainable Agro-ecosystems and Bioresources, Research and Innovation Centre, Fondazione Edmund Mach, Via E. Mach 1, 38010 San Michele all’Adige, Italy; 11 Institute of Agricultural and Environmental Sciences, Estonian University of Life Sciences, Kreutzwaldi 5, 51014 Tartu, Estonia; 12 Helmholtz Centre for Environmental Research, Department of Lake Research and Department of Aquatic Ecosystem Analysis, Magdeburg, Germany; 13 CNR Water Research Institute, L.go Tonolli 50, I-28922 Verbania Pallanza, Italy; 14 Dorset Environmental Science Centre, Dorset, ON P0A 1E0 Canada; 15 Department of Aquatic Ecology and Environmental Biology, Institute for Water and Wetland Research, Radboud University, 6525AJ Nijmegen, The Netherlands; 16 Department of Limnology and Biological Oceanography, University of Vienna, Althanstrasse 14, 1090 Vienna, Austria; 17 IISD Experimental Lakes Area Inc., 111 Lombard Avenue Suite 325, Winnipeg, R3B 0T5 Canada; 18 U.S. Geological Survey, New York Water Science Center, Troy, NY 12180 USA; 19 Centre for Limnology, Institute of Agricultural and Environmental Sciences, Estonian University of Life Sciences, 51117 Rannu, Tartu County Estonia; 20 Institute of Biology, University of Latvia, Miera Str.3, Salaspils, LV-2169 Latvia; 21 U.S. Environmental Protection Agency, Clean Air Markets Division, Washington, DC 20460 USA; 22 Vermont Department of Environmental Services, 1 National Life Drive, Montpelier, Vermont USA; 23 Irstea, RiverLy, 5 Rue de la Doua -, 69625 Villeurbanne cedex, France; 24 Norwegian Institute for Water Research, Gaustadalléen 23, NO-0349 Oslo, Norway; 25 Biological Station Lake Neusiedl, 7142 Illmitz, Austria

**Keywords:** Freshwater ecology, Element cycles, Environmental impact

## Abstract

Calcium (Ca) is an essential element for almost all living organisms. Here, we examined global variation and controls of freshwater Ca concentrations, using 440 599 water samples from 43 184 inland water sites in 57 countries. We found that the global median Ca concentration was 4.0 mg L^−1^ with 20.7% of the water samples showing Ca concentrations ≤ 1.5 mg L^−1^, a threshold considered critical for the survival of many Ca-demanding organisms. Spatially, freshwater Ca concentrations were strongly and proportionally linked to carbonate alkalinity, with the highest Ca and carbonate alkalinity in waters with a pH around 8.0 and decreasing in concentrations towards lower pH. However, on a temporal scale, by analyzing decadal trends in >200 water bodies since the 1980s, we observed a frequent decoupling between carbonate alkalinity and Ca concentrations, which we attributed mainly to the influence of anthropogenic acid deposition. As acid deposition has been ameliorated, in many freshwaters carbonate alkalinity concentrations have increased or remained constant, while Ca concentrations have rapidly declined towards or even below pre-industrial conditions as a consequence of recovery from anthropogenic acidification. Thus, a paradoxical outcome of the successful remediation of acid deposition is a globally widespread freshwater Ca concentration decline towards critically low levels for many aquatic organisms.

## Introduction

Calcium (Ca) accounts for less than 3% of the weight of most living organisms^1^. Although this proportion is small, Ca is considered an essential element for the growth and population dynamics of freshwater flora and fauna by influencing intracellular signalling, neuron activity, muscle contraction, and enzymatic processes^2,3^. Ca is also important as a key structural component for invertebrates with a calcified exoskeleton as well as for bony structures in vertebrates and egg-shell formation in birds^2^. Several studies suggest that Ca concentration is an important driver of community structure in freshwaters^4,5^. An example of a distinctive community change, which has been attributed to declining Ca concentration in Canadian lakes, is the observed replacement of the Ca-demanding cladoceran *Daphnia* spp. by the Ca-poor cladoceran *Holopedium glacialis*, a process known as jellification^6^. Changes in freshwater community composition in response to changing Ca availability reflect the differential Ca requirements of freshwater biota^7,8^.

A variety of freshwaters, particularly those in the Canadian Shield and in Fennoscandia, presently have Ca concentrations that are critically low for growth, reproduction and survival of Ca-demanding organisms such as mussels, snails, crustacean zooplankton and crayfish e.g.^2,9–11^, and which most likely also affect a suite of other organisms, ranging from phytoplankton^12^ to fish^13^ and birds^14^. Although measurements of Ca concentrations are available for major rivers throughout the world e.g.^15^ they are comparably rare for the 117 million lakes on Earth^16^, resulting in a knowledge gap of how widespread critically low Ca concentrations are for the reproduction and survival of a large variety of Ca-demanding organisms in freshwaters.

A major natural source of Ca to freshwaters is mineral weathering^17^. Soils and rocks in the catchments of water bodies can contain substantial amounts of calcium-bearing minerals like calcium carbonate (CaCO_3_), which reacts with carbonic acid (H_2_CO_3_) from atmospheric and soil respiratory sources^17^. When H_2_CO_3_ reacts with CaCO_3_, the ions Ca^2+^, bicarbonate (HCO_3_^−^) and carbonate (CO_3_^2−^) are produced, and they are transported into freshwaters, eventually reaching the oceans^18^. The sum of the charges of the dissolved species HCO_3_^−^ and CO_3_^2−^ is referred to as carbonate alkalinity, often expressed in charge equivalents per liter.

Bicarbonate and carbonate are species of the carbonate system, a system that regulates the pH of water and controls the cycling of CO_2_ between the biosphere, lithosphere, atmosphere and oceans^19^. The carbonate system has been described as one of the most ancient global biogeochemical systems on Earth, being essential for all biological systems^20^. Due to the importance of the carbonate system, substantial research efforts have been made to predict spatial and temporal variation in carbonate species^21,22^. In this context, close interactions between the carbon and the Ca cycles in the ocean-atmosphere system have been identified^23^.

**Figure 1:**
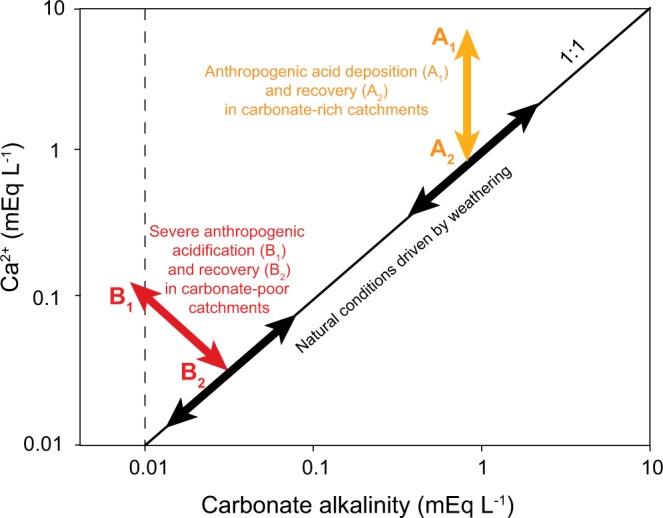
Conceptual figure showing the influence of anthropogenic acidification on the relationship between dissolved calcium (Ca^2+^) and carbonate alkalinity in freshwaters. Under natural conditions, Ca^2+^ and carbonate alkalinity are expected to co-vary in a relative charge-equivalent proportion (1:1 proportion in figure), with catchment-specific concentrations reflecting the weathering potential of the catchment’s bedrock and soils, and thermodynamic limits (black arrows). Under the influence of anthropogenic acidification, the carbonate buffering system of soils and water bodies can become depleted and cations such as Ca^2+^ increasingly leach from soils into freshwaters. Depending on the buffering capacity of catchment soils, acidification causes the Ca^2+^ excess in relation to carbonate alkalinity to follow the direction of arrow A_1_ in case of a high buffering capacity, and the direction of arrow B_1_ in case of a low buffering capacity. In both cases, the 1:1 charge-equivalent proportion is disturbed. Freshwaters can even reach negative alkalinity (left of dashed line) due to high concentrations of free hydrogen ions. When catchments and freshwaters recover from anthropogenic acidification, the above described process is reversed (arrows A_2_ and B_2_, respectively).

Although lakes are known to play an important role in the global carbon cycle^24,25^, interactions between Ca and carbonate species have so far not been properly accounted for in a global context. Lakes usually have a pH between 6 and 8, a pH range where the bicarbonate ion dominates relative to the carbonate ion^26^. Conceptually, and assuming that weathering is the main source and driver, Ca and carbonate alkalinity are expected to show a positive co-variation in lakes as well as running waters (Fig. 1). Under certain conditions, however, such as with additions of strong acid anions arising from anthropogenic acidification (particularly sulfate), the carbonate buffering system (i.e. alkalinity) of soils and water bodies becomes depleted and positive ions begin to leach from soils into waters without being counterbalanced by alkalinity. The leaching of positive ions, including Ca^2+^, from soils into waters under the influence of acidification is well described in the literature^27,28^. Thus, one result of anthropogenic acidification is an expected excess of Ca in relation to carbonate alkalinity in freshwaters (Fig. 1).

The Ca excess induced by anthropogenic acidification is reversible when soils and freshwaters recover from acidification (Fig. 1). In response to mitigation of emissions of acid precursors to the atmosphere, many freshwaters in the Northern Hemisphere are presently in such a recovery phase^29^, and thus they are expected to show a rapid decline in Ca concentrations until natural conditions have been re-established with a charge-equivalent proportion between Ca^2+^ and carbonate alkalinity (Fig. 1). Such a rapid Ca concentration decline has in fact been observed in a variety of freshwaters^2,11,30^. In those studies, however, focus has been on Ca concentration declines only and not on the relationship to carbonate alkalinity concentrations. Therefore, it remains unknown for how long the process of declining Ca concentrations will continue into the future, and how many freshwaters might reach critically low Ca levels for the survival and reproduction of the most sensitive organisms. According to recent studies there is even a risk that Ca concentrations may fall below pre-industrial levels due to the historical depletion of base cation stores in the catchments of acid-sensitive regions^31^, and/or the removal of Ca via timber harvesting^32^. If such additional Ca depletion processes occur, critically low Ca levels might even become more widespread than predicted by the conceptual model (Fig. 1).

Since globally many regions have historically been affected by anthropogenic acidification and the recovery is still ongoing^29^ we hypothesized that many freshwaters around the globe still show an excess of Ca over carbonate alkalinity concentrations, but that most freshwaters are returning to pre-industrial (or even below) Ca and carbonate alkalinity concentrations. To test the hypothesis, we present here a global analysis of Ca and carbonate alkalinity concentrations in freshwaters (i.e., lakes including a few large lakes and reservoirs as well as rivers and streams). Our study provides new insights into the global distribution and drivers of Ca concentrations in freshwaters, which has relevance not only for freshwater biota, but also for the global carbonate cycle.

## Methods

### Data and variables

**Figure 2:**
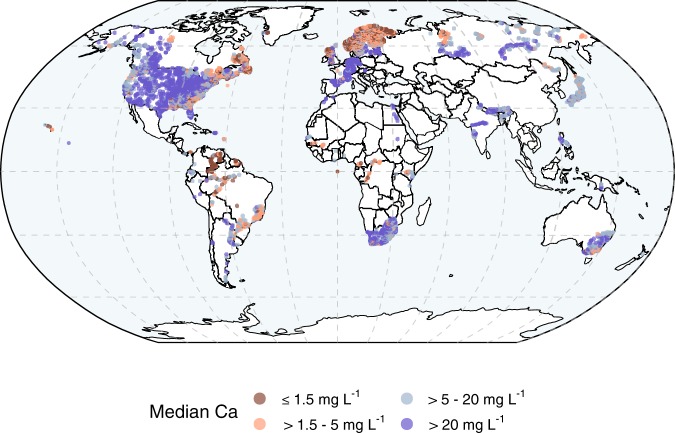
Global distribution of long-term median calcium (Ca) concentrations in lakes and running waters. In dark red are site-specific long-term median Ca concentrations ≤ 1.5 mg HCO_3_^−^ L^−1^ representing an approximate threshold considered critical for the reproduction and survival of a large variety of aquatic organisms. Some available long-term median Ca concentrations could not be shown in the figure, mainly from sites located in North America and Fennoscandia, due to the lack of exact geographical locations of sampling points.

We collected data on Ca concentrations, bicarbonate and carbonate concentrations (or total dissolved inorganic carbon concentrations), pH and alkalinity from 43 184 lake, reservoir, river and stream sites around the globe (Fig. 2, Supplementary Table 1), comprising a total of 440 599 water samples from 57 countries. From the 440 599 water samples, 279 940 samples were from rivers and streams and 160 659 from lakes including 37 106 samples from large lakes and reservoirs. The data were provided by members of the Global Lake Ecological Observatory Network (GLEON, http:www.gleon.org) and from the global river chemistry database^33^ (GLORICH: https://doi.pangaea.de/10.1594/PANGAEA.902360). Data on Ca concentrations were available in mg L^−1^, mEq L^−1^ or mmol L^−1^, most of them being measured as dissolved Ca^2+^. To make our data comparable, we transferred all data to mg L^−1^ according to http://unitslab.com/node/43. Whenever we refer to mg L^−1^ we use the abbreviation Ca. In some cases, consideration of charges became important. Whenever we refer to charges, we use the unit mEq L^−1^ and the abbreviation Ca^2+^.

Altogether we had 440 599 data points on Ca concentrations, 432 283 data points on pH and 284 690 data points on carbonate alkalinity (Supplementary Table 1). Carbonate alkalinity in mEq L^−1^ was determined either as the sum of bicarbonate and carbonate ions (for data from the global river chemistry database and data from Lake Neusiedl, central Europe) or as dissolved inorganic carbon concentrations minus the amount of free CO_2_ calculated from alkalinity, pH and water temperature^34^. For comparison with Ca concentrations in mg L^−1^ the alkalinity concentrations were converted to mg HCO_3_^−^ L^−1^ according to http://unitslab.com/node/92. Due to large uncertainties in calculated CO_2_ concentrations in highly acidic, dissolved organic carbon-rich waters^35^ we only used positive alkalinity measurements, resulting in a lack of alkalinity data from the most acidic waters. Complete data on carbonate alkalinity, Ca and pH were available for 21 902 lakes and 16 339 running waters. Most lakes and running waters had multiple measurements from different seasons and different years. For some analyses we used long-term site-specific median values, which we calculated as the overall median of the annual medians for each lake or running water site.

For evaluation of temporal changes, we analyzed complete time series (at least four measurements per year) of Ca and pH (available from 297 freshwaters), carbonate alkalinity (available from 211 freshwaters) and sulfate concentrations (available from 213 freshwaters; here used as a proxy for the influence of anthropogenic acidification) from 1980 to 2017 (Table 1). For trend analyses, we used site-specific yearly median values.

**Table 1:** Results of Mann-Kendall trend tests of calcium (Ca) concentrations from 296 freshwaters since 1980 and number of significant relationships (*p* < 0.05) between Ca and carbonate alkalinity (Ca~Alk) and between Ca and sulfate (SO_4_^2−^) concentrations (Ca~SO_4_) on a temporal scale, using yearly median values. The abbreviation n.a. reflects non-available data.

Country	Site	Long-term median pH	Length of available time series	Number of sites with significant Ca trends	% of significantly decreasing Ca trends	Number of sites confirming positive Ca~Alk	Number of sites showing positive Ca~SO_4_
**Countries/regions with waters that showed significantly declining Ca concentration trends**							
Czech Republic	4 inland waters	6.9–7.4	1980/1983–2017	4 out of 4 waters (all−)	100	0 out of 4 waters	4 out of 4 waters
Germany	Rappbode Reservoir	7.5	1980–2016	1 out of 1 (all−)	100	n.a.	1 out of 1
Switzerland	Lake Zurich	8.4	1987–2017	1 out of 1 (all−)	100	1 out of 1	0 out of 1
Netherlands	5 inland waters	4.3–7.7	1980/1991–2016/2017	3 out of 5 (all−)	60	0 out of 5 waters	n.a.
Sweden	66 inland waters	6.3–7.9	1980–2017	39 out of 66 (34−, 5+)	52	40 out of 66	42 out of 66
Norway	77 inland waters	4.6–6.6	1986–2016	50 out of 77 (33−, 17+)	43	13 out of 77	39 out of 77
US Adirondacks	44 lakes	4.3–6.9	1992/1993–2016/2017	42 out of 44 (all−)	96	8 out of 44	43 out of 44
US East Coast	59 inland waters	4.4–6.9	1980/1994–2016	31 out of 59 (38−, 3+)	48	5 out of 52 waters (n.a. for 7 sites)	32 out of 59 waters
Eastern Canada	8 Dorset lakes, 1 ELA lake	5.6–7.0	1980–2015	8 out of 9 lakes (all−)	89	5 out of 8 lakes (n.a. for ELA)	7 out of 8 lakes (n.a. for ELA)
**Countries/regions with waters that showed no or significantly increasing Ca concentration trends**							
Estonia/Russia	Lake Peipsi	8.3	1996–2017	2 out of 5 sites (all+)	0	3 out of 5 sites	0 out of 5 sites
Estonia	Lake Vörtsjärv	8.3	1989–2017	0 out of 1	0	1 out of 1	0 out of 1
Latvia	14 inland waters	3.9/7.8–8.1	1980–2017	1 out of 14 waters (all+)	0	10 out of 14 waters	8 out of 14 waters
Austria/Hungary	Lake Neusiedl	8.8	1987–2017	0 out of 4 sites	0	0 out of 4 sites	0 out of 4 sites
France/Switzerland	Lake Geneva	8.4	1980–2016	0 out of 1	0	1 out of 1	0 out of 1
France	Upper River Loire	7.5	1980–2015	2 out of 2 sites (all+)	0	2 out of 2 sites	0 out of 2 sites
Italy	Lake Mergozzo, Lake Paione Superiore	6.0–7.0	1984–2016	0 out of 2 lakes	0	1 out of 2 lakes	1 out of 2 lakes
Italy	Lake Caldonazzo	8.3	1980–2014	1 out of 1 (all+)	0	1 out of 1	n.a.
US	Gull Lake, Michigan	8.2	1996–2017	0 out of 1	0	1 out of 1	0 out of 1

### Statistics

All statistical tests were carried out in the statistical program JMP, version 12.0 (SAS Institute Inc.). Because of non-normal distributions of our data we used non-parametric statistical methods such as the Wilcoxon test for group comparisons and Kendall’s tau for correlation analyses. Boxplots are normal quantile boxplots with 25^th^ and 75^th^ percentiles, and an upper quartile + 1.5 * (interquartile range) and a lower quartile – 1.5 * (interquartile range). The significance of temporal trends was determined by applying the non-parametric Mann-Kendall trend test to yearly median values with a significance level of *P* < 0.05.

## Results and Discussion

### Spatial distribution of freshwater Ca concentrations

**Figure 3:**
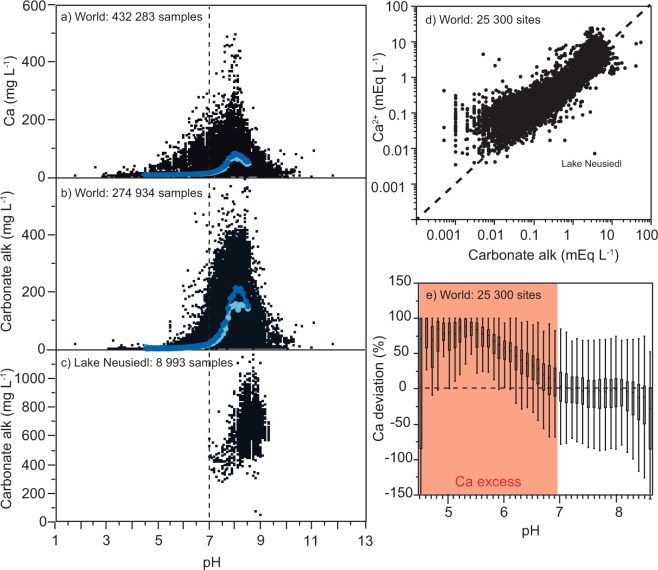
Calcium (Ca), pH and carbonate alkalinity of 43 184 lake and running water sites. Panel a shows all available Ca concentration data and panel b shows all available carbonate alkalinity (carbonate alk) data in relation to pH except data from Lake Neusiedl in central Europe, which is shown separately (panel c) because this lake is an endorheic basin with disproportionally high carbonate alkalinity in relation to Ca concentrations (panel d). Panels a and b show the median Ca concentration and carbonate alkalinity for each 0.1 pH unit, based on site-specific long-term median values for running waters (dark blue dots) and lakes (light blue dots). The median values as well as other percentiles are available in Supplementary Table 2. Panel d shows the relationship between Ca^2+^ and carbonate alkalinity, based on site-specific long-term median values for all freshwater sites. On a global scale, Ca^2+^ and carbonate alkalinity generally follow a 1:1 relation (dotted line). However, systematic deviations from a 1:1 relation occur, displayed as percentage for each pH unit in panel e.

Analyzing all available Ca data, sampled during different seasons and across lakes and running waters distributed over 57 countries (altogether 440 599 measurements), we found that concentrations varied between 0.4 and 74.4 mg L^−1^ (2.5 and 97.5 percentiles, respectively) with a median of 4.0 mg L^−1^ (median of 279 940 running water samples: 3.76 mg L^−1^ and median of 160 659 lake samples: 5.50 mg L^−1^). The lowest Ca concentrations occurred mainly in the boreal region, i.e. Fennoscandia and eastern Canada, but also in the United Kingdom, some regions in eastern North America and in some subtropical and tropical regions of South America (Fig. 2). Freshwater Ca concentrations on a global scale showed a clear right-skewed distribution with only a few freshwater samples having Ca concentrations > 450 mg L^−1^ (Fig. 3a). These high-Ca freshwater samples had a large influence on the global mean Ca concentration of 12.5 mg L^−1^ (mean of 279 940 running water samples: 11.12 mg L^−1^ and mean of 160 659 lake samples: 14.95 mg L^−1^), making it comparable to the previously reported mean Ca concentration of the world river input to oceans of about 14 mg L^−1^ ^15^.

The large discrepancy between the global mean and median Ca concentrations in freshwaters reflects the uneven distribution of Ca concentrations in the world where freshwaters with Ca concentrations ≤ 4.0 mg L^−1^ predominate, with 20.7% of the water samples showing Ca concentrations ≤ 1.5 mg L^−1^, a threshold considered critical for the reproduction and survival of a variety of aquatic organisms^4^. Ca concentrations are often naturally low because of regional geology (e.g. gneissic or granitic bedrocks), highly weathered soils (as in much of the tropics), and/or limited weathering potential in the catchment (mainly due to a cold climate)^36–38^. Low Ca concentrations can also be exacerbated by anthropogenic drivers, such as timber harvesting e.g.^32,39^ and recovery from historical acidification e.g.^2,27^. In our global dataset we found critically low Ca concentrations ≤ 1.5 mg L^−1^ in nutrient-poor, acidic boreal waters with a pH ≤ 5.5 (Fig. 3a and Supplementary Information). These waters were mainly located in regions that commonly have a limited weathering potential in the catchment due to seasonal ice cover and generally rather low annual mean air temperatures^37^ (Fig. 2). In addition, these waters have historically been exposed to anthropogenic acid deposition, and their catchments have been depleted in base cations^40,41^. Some low concentrations of Ca were also observed in lowland tropical and subtropical regions (Fig. 2), where soils tend to be much older compared to other geographical regions and therefore often are depleted of readily weatherable carbonate minerals^11,13^.

### Comparison of Ca concentrations between lakes and running waters

On a global scale, we observed that Ca concentrations in lakes and running waters gradually increased along a pH gradient, up to a pH of around 8.0 (Fig. 3a). The Ca increase along the pH gradient was similar between lakes and running waters (Fig. 3a and Supplementary Table 2). However, despite similarities between lentic and lotic waters, we found significantly higher Ca concentrations in our running waters than lakes along the entire pH gradient (non-parametric Wilcoxon test for 42 different pH categories within the range 4.5–8.6: *p* < 0.05 for 40 out of 42 pH categories; Supplementary Table 2). Lower Ca concentrations in lakes than in running waters might simply reflect that the majority of the lake sites considered in this study were located in relatively cold geographical regions with a limited weathering potential, while our running water sites were more equally distributed across latitudes. There are, however, other reasons why Ca concentrations in lakes can be lower than in running waters. In the more precipitation-fed lakes, Ca originating from the catchments and entering via groundwater and stream inputs is often significantly diluted by the direct capture of precipitation onto the lake surface. Lakes also have a substantial internal loss of Ca caused by various biotic and abiotic processes, such as phyto- and zooplankton uptake and subsequent sedimentation^42^ and CaCO_3_ precipitation and sedimentation in alkaline lakes e.g.^43,44^. CaCO_3_ supersaturation and precipitation commonly occur at pH > 8.0, which likely explains why we found no further Ca concentration increases above this pH (Fig. 3a).

### Ca concentrations and carbonate alkalinity

Ca and carbonate alkalinity concentrations showed a similar pattern along the pH gradient, with maximum concentrations in the range of pH 8.0–9.0 (Fig. 3b). The observed pattern along the pH gradient followed the theoretical dissolved inorganic carbon speciation in natural waters^45^. Taking the long-term surface-water median value for each site, we found that Ca and carbonate alkalinity strongly co-varied (Kendall’s tau: *p* < 0.0001), approximately following a 1:1 relation (Fig. 3d). Thus, we found that Ca and carbonate alkalinity are generally in balance, suggesting that their concentrations are controlled by similar drivers, particularly carbonate mineral weathering, which supports our conceptual model (Fig. 1). At high pH, i.e. in hardwater lakes, we suggest that Ca and carbonate alkalinity remain in balance due to CaCO_3_ precipitation as a sink.

Despite the overall Ca and carbonate alkalinity co-variation across the range of freshwater concentrations, we observed hydrology- and pH-related deviations from this general pattern. We found that isolated, saline waters, e.g. the endorheic Lake Neusiedl in central Europe, can substantially deviate from a Ca:alkalinity ratio of 1:1. In Lake Neusiedl, where carbonate alkalinity is disproportionally high in relation to Ca (Fig. 3d), the excess alkalinity is largely balanced by sodium.

In addition to endorheic saline lakes, waters with pH < 7.0 also could show deviations from a Ca:alkalinity ratio of 1:1. In these waters we observed a Ca excess in relation to carbonate alkalinity that gradually increased with decreasing pH (Fig. 3e), suggesting that acid anions other than bicarbonate become relevant in the ion balance. One important ion associated with acidification is sulfate, which is characteristic of acid deposition from the atmosphere, but can also originate from fertilizers^46^ and acid mine drainage^47^, as well as natural mineral deposits (e.g., oxidation of iron sulfides). Sulfate can cause an accelerated leaching of Ca from soils into freshwaters by decreasing the soil pH and thereby triggering Ca displacement by the hydrogen ion in the soil sorption complex^46,48,49^. Since we found the Ca excess in relation to carbonate alkalinity mainly in waters located in regions that historically have been exposed to acid deposition^40^ (i.e. eastern North America, eastern Europe and Fennoscandia), we infer that acid deposition was the ultimate driver decoupling the relationship between Ca and carbonate alkalinity, consistent with our conceptual model (Fig. 1). Accordingly, Ca concentrations in regions that are presently recovering from acidification are expected to return towards pre-industrial conditions where Ca and carbonate alkalinity are strongly coupled and in balance, and Ca concentrations are lower than they were at the peak of catchment acidification (Fig. 1). Post-acidification recovery might even drive Ca concentrations below pre-acidification concentrations due to a depletion of base cation stores in the catchment soils of acid-sensitive regions^31^.

### Ca concentration changes over time

We tested our expectation of a return to stoichiometrically balanced Ca and carbonate alkalinity in freshwaters by analyzing time series of Ca, carbonate alkalinity, pH and sulfate from 1980 to 2017. Altogether we analyzed complete time series of Ca and pH from 297 freshwaters, including carbonate alkalinity from 211 freshwaters from 1980 to 2017 (see Methods). Almost all freshwaters with available time series were located in regions that have historically been exposed to acid deposition^40^, and thus all of them could potentially demonstrate declining Ca concentrations (Fig. 1). We found that out of the 297 freshwaters more than half (164 sites) showed significantly decreasing Ca concentrations since 1980 (Table 1). In waters with significantly decreasing Ca concentrations, Ca no longer showed a positive relationship to carbonate alkalinity, implying that Ca concentrations have decreased despite constant or increasing carbonate alkalinity. Instead of being coupled to the temporal change in carbonate alkalinity, Ca concentrations showed a very strong positive relationship to the temporal change in sulfate concentrations (Table 1).

**Figure 4:**
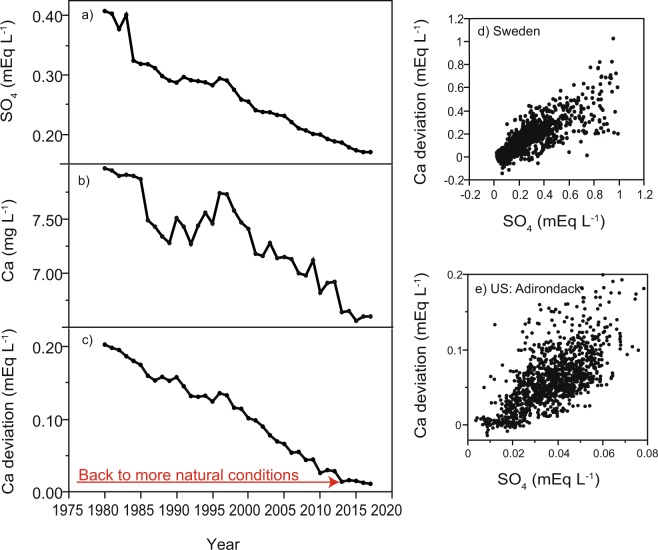
Temporal variation in freshwater chemistry. Shown are temporal variations in median sulfate concentration (SO_4_^2−^, panel a), calcium concentration (Ca, panel b), and Ca^2+^ deviation from the 1:1 relationship between Ca^2+^ and carbonate alkalinity (panel c) at the mouth of the Göta älv at Trollhättan, the Swedish river with the largest Ca decline from 1980 to 2017. The Ca^2+^ deviation from a 1:1 relation to carbonate alkalinity was further determined for 66 inland waters in Sweden and 44 lakes in the US Adirondacks, all located in regions that have historically been exposed to acid deposition. Panels d and e show the relationship between yearly median SO_4_^2−^ concentrations and the Ca^2+^ deviation from a 1:1 relation to carbonate alkalinity during 1980 to 2017. The relationships are highly significant (Kendall’s tau correlation: *p* < 0.001) for both Swedish and US freshwaters.

A coherent decline in sulfate and Ca concentrations over time was especially apparent in a river mouth in southern Sweden, which demonstrated the most significant Ca concentration decline in Sweden since 1980 (Fig. 4a,b). The coherent sulfate and Ca decreases corresponded to a return of Ca concentrations to an approximate charge balance with carbonate alkalinity (Fig. 4c). We suggest that acidification associated with anthropogenic sulfate sources is an important driver of the displacement from natural conditions where Ca and carbonate alkalinity are strongly coupled and in balance. Our suggestion is supported by region-specific patterns for freshwaters located in two geographical regions that are well known to have recovered from historical acidification—the US Adirondacks and Sweden^30,50^. In these two regions, we observed a highly significant positive relationship between sulfate concentrations and Ca deviation from the 1:1 ratio between Ca and carbonate alkalinity, with the vast majority of data showing a Ca excess (Fig. 4d,e). Thus, our results provide evidence that acidification caused the decoupling between Ca and carbonate alkalinity across large regions of the globe via a selective sulfate-driven Ca leaching from catchment soils into freshwaters. In many regions, acid deposition has now been mitigated and the Ca-alkalinity coupling is becoming re-established.

Despite being in regions that have been exposed to acid deposition, 45% of the freshwaters in our dataset did not show a statistically significant decline in Ca concentrations over recent decades (Table 1). There are many possible reasons to explain this pattern: (a) liming and other management activities have occurred in the catchment e.g.^51,52^, (b) the critical load of acidity for surface waters by inputs of acids from acid deposition did not exceed the natural weathering potential^53^, (c) waters have already recovered from acidification, reaching a balance with carbonate alkalinity according to the conceptual model (Fig. 1), (d) mineral weathering rates have increased due to climate change e.g.^54,55^, (e) there has been an increased use of Ca in CaCl_2_ road salt^56^ and for soil liming in agricultural areas^46^, (f) there has been an increase in concentrations of dissolved organic carbon and its associated cations such as Ca, and (g) available intra-annual measurements were too infrequent and time-series too short to detect an existing trend with statistical significance.

In most of the waters in which Ca concentrations did not significantly decline, we found a strong positive relationship between Ca and carbonate alkalinity but not between Ca and sulfate concentrations (Table 1). These results suggest that although drivers for Ca concentrations in lakes and running waters might vary substantially, Ca and carbonate alkalinity generally exist in stoichiometrically balanced proportions. We found, however, that sulfate has the potential to induce strong deviations in the global co-variation between Ca and carbonate alkalinity, presumably reflecting strong mineral acidity associated with sulfate sources. Since anthropogenic sulfate is not only associated with acid precipitation but also with fertilizers and acid mine drainage, the consequences of widespread sulfate pollution need to be carefully evaluated.

## Conclusion

Our global analysis of Ca and carbonate alkalinity concentrations in lakes and running waters shows that Ca and carbonate alkalinity generally strongly co-vary with highest concentrations in waters with a pH between 8.0 and 9.0, and lowest concentrations in acidic water bodies. Under the influence of anthropogenic acid deposition, however, Ca concentrations became disproportionate and unnaturally high relative to alkalinity, often accompanied by higher sulfate concentrations. As acid deposition has been increasingly mitigated in North America and Europe and freshwaters recover from anthropogenic acidification, Ca concentrations rapidly decline towards a state where they again become balanced with carbonate alkalinity. Ca concentrations may even decline below pre-acidification concentrations due to a depletion of base cations stores in the catchment soils of acid-sensitive regions, or because of other stressors including timber harvesting. Since Ca concentrations are generally low in many freshwaters, in some regions critically low, further Ca concentration declines as freshwaters fully recover from acidification will likely have widespread consequences for biota and ecosystem processes.

## Supplementary information

Supplementary Information
