# Supplementary material for: Widespread diminishing anthropogenic effects on calcium in freshwaters
Source: Sci Rep. 2019 Jul 18;9:10450. doi: 10.1038/s41598-019-46838-w (PMC6639332; doi:10.1038/s41598-019-46838-w)
Supplement: Supplementary file 1 — Supplementary Information [file 41598_2019_46838_MOESM1_ESM.docx]

**Supplementary Information**

**Title: Widespread diminishing anthropogenic effects on calcium in freshwaters**

Gesa A. Weyhenmeyer^1,*^, Jens Hartmann^2^, Dag O. Hessen^3^, Jiří Kopáček^4^, Josef Hejzlar^4^, Stéphan Jacquet^5^, Stephen K. Hamilton^6^, Piet Verburg^7^, Taylor H. Leach^8^, Martin Schmid^9^, Giovanna Flaim^10^, Tiina Nõges^11^, Peeter Nõges^11^, Valerie C. Wentzky^12^, Michela Rogora^13^, James A. Rusak^14^, Sarian Kosten^15^, Andrew M. Paterson^14^, Katrin Teubner^16^, Scott N. Higgins^17^, Gregory Lawrence^18^, Külli Kangur^19^, Ilga Kokorite^20^, Leonardo Cerasino^10^, Clara Funk^21^, Rebecca Harvey^22^, Florentina Moatar^23^, Heleen A. de Wit^24^, Thomas Zechmeister^25^

*^1^Department of Ecology and Genetics/Limnology, Uppsala University, Norbyvägen 18D, 752 36 Uppsala, Sweden*

*^2^Department of Earth Sciences, University of Hamburg, Bundesstraße 55, 20146 Hamburg, Germany*

*^3^University of Oslo, Dept. Biosciences, Centre for Biogeochemistry in the Anthropocene (CBA), Box 1066 Blindern, 0316, Norway*

*^4^Institute of Hydrobiology, Biology Centre CAS, Na Sádkách 7, 370 05 České Budějovice, Czech Republic*

*^5^INRA CARRTEL, 75 bis avenue de Corzent, 74203 Thonon-les-Bains cx, France*

*^6^Kellogg Biological Station and Dept. Integrative Biology, Michigan State University, Hickory Corners, MI 49060 and Cary Institute of Ecosystem Studies, Millbrook, NY 12545 USA*

*^7^National Institute of Water and Atmospheric Research, Hamilton, New Zealand*

*^8^Department of Biological Sciences, Rensselaer Polytechnic Institute, Troy, NY 12180, USA*

*^9^Surface Waters – Research and Management, Eawag: Swiss Federal Institute of Aquatic Science and Technology, Seestrasse 79, 6047 Kastanienbaum, Switzerland*

*^10^Department of Sustainable Agro-ecosystems and Bioresources, Research and Innovation Centre, Fondazione Edmund Mach, Via E. Mach 1, 38010 San Michele all'Adige, Italy*

*^11^Institute of Agricultural and Environmental Sciences, Estonian University of Life Sciences, Kreutzwaldi 5, 51014 Tartu, Estonia*

*^12^Helmholtz Centre for Environmental Research, Department of Lake Research and Department of Aquatic Ecosystem Analysis, Magdeburg, Germany*

*^13^CNR Water Research Institute, L.go Tonolli 50. I-28922 Verbania Pallanza, Italy*

*^14^Dorset Environmental Science Centre, Dorset, ON, Canada P0A 1E0*

*^15^Department of Aquatic Ecology and Environmental Biology, Institute for Water and Wetland Research, Radboud University, 6525AJ Nijmegen, The Netherlands*

*^16^Dept. of Limnology and Biological Oceanography, University of Vienna, Althanstrasse 14, 1090 Vienna, Austria*

*^17^IISD Experimental Lakes Area Inc., 111 Lombard Avenue Suite 325, Winnipeg R3B 0T5, Canada*

*^18^U.S. Geological Survey, New York Water Science Center, Troy, NY, 12180, USA*

*^19^Centre for Limnology, Institute of Agricultural and Environmental Sciences, Estonian University of Life Sciences, 51117 Rannu, Tartu County, Estonia*

*^20^Institute of Biology, University of Latvia, Miera Str.3, Salaspils, LV-2169, Latvia*

*^21^U.S. Environmental Protection Agency, Clean Air Markets Division, Washington, DC 20460, USA*

*^22^Vermont Department of Environmental Services, 1 National Life Drive, Montpelier, Vermont, USA*

*^23^Irstea, RiverLy, 5 Rue de la Doua - 69625 Villeurbanne cedex, France*

*^24^ Norwegian Institute for Water Research, Gaustadalléen 23, NO-0349 Oslo, Norway*

*^25^Biological Station Lake Neusiedl, 7142 Illmitz, Austria*

* *Corresponding author, e-mail:* [*Gesa.Weyhenmeyer@ebc.uu.se*](mailto:Gesa.Weyhenmeyer@ebc.uu.se)

**Supplementary Table 1**. Number of available water samples on calcium (Ca), pH and carbonate alkalinity from various countries and freshwaters. Transboundary lakes and rivers were allocated to the country from where the data were received.

| Country | Sites | Number of samples | | | Data source |
| --- | --- | --- | --- | --- | --- |
|  |  | Ca | pH | Carbonate alkalinity |  |
| World rivers | 18 897 river sites | 12 120 | 11 443 | 8 083 | Glorich (J. Hartmann)^1^ |
| Austria | 1 lake, 38 sites | 9 388 | 9 288 | 8 993 | K. Teubner, T. Zechmeister |
| Canada | 9 lakes | 311 | 311 | 0 | J. Rusak, A. Paterson, S. Higgins |
| Czech Rep. | 6 inland waters | 4 279 | 4 279 | 3 165 | J. Hejzlar, J. Kopáček |
| Estonia | 2 lakes, 6 sites | 640 | 615 | 603 | T. Nõges, P. Nõges^2^, K. Kangur |
| Finland | 873 lakes | 873 | 873 | 0 | D. Hessen^3^ |
| France | 572 inland waters | 117 095 | 115 830 | 95 536 | F. Moatar^4^, S. Jacquet^5^ |
| Germany | 1 reservoir | 3 170 | 3 161 | 0 | V. Wentzky |
| Italy | 13 inland waters | 2 533 | 2 530 | 2 065 | G. Flaim, L. Cerasino, M. Rogora^6^ |
| Latvia | 21 running waters | 4 327 | 4 285 | 4 159 | I. Kokorite^7^ |
| Netherlands | 8 inland waters | 2 476 | 2 111 | 0 | S. Kosten^8^ |
| New Zealand | 1 river | 40 | 40 | 0 | P. Verburg |
| Norway | 1 371 inland waters | 4 302 | 3 958 | 0 | D. Hessen, H. de Wit^9^ |
| Russia | 293 inland waters | 489 | 487 | 0 | D. Hessen |
| Sweden | 20 883 inland waters | 243 994 | 242 396 | 159 861 | G. Weyhenmeyer^10^ |
| Switzerland | 1 lake | 7 538 | 7 535 | 0 | M. Schmid^11^ |
| UK | 188 inland waters | 188 | 188 | 0 | D. Hessen |
| US | 574 inland waters | 26 836 | 22 953 | 2 225 | T. Leach, G. Lawrence, R. Harvey, C. Funk^12^, S. Hamilton |
| Total | 43 184 sites | 440 599 | 432 283 | 284 690 |  |

^1^Data are available at <https://www.geo.uni-hamburg.de/en/geologie/forschung/geochemie/glorich.html>

^2^Data are from the Estonian Environment Agency

^3^Data are from the Nordic lake inventory program

^4^Data are from the French national monitoring network

^5^Data from the French-Swiss Lake Geneva were collected by OLA observatory, with thanks to INRA Thonon-les-

Bains, and CIPEL. The SOERE OLA is developed by ORE Eco-Informatique group.

^6^Data have been provided by LTER (Long-Term Ecological Research Network) and CIPAIS (International

Commission for the Protection of Waters between Italy and Switzerland)

^7^Data have been provided by the Latvian Environmental, Geology and Meteorology Centre

^8^Data of the rivers Rhine, Meuse and Lake Ijssel are available at

[https://www.rijkswaterstaat.nl/water/waterdata-en-](https://www.rijkswaterstaat.nl/water/waterdata-en-   waterberichtgeving/waterdata)

[waterberichtgeving/waterdata](https://www.rijkswaterstaat.nl/water/waterdata-en-   waterberichtgeving/waterdata); data from moorland pools are from J.G.M. Roelofs

^9^Data are from the Nordic and the Norwegian freshwater inventory program

^10^Data are from the Swedish freshwater inventory program, available at <https://www.slu.se/en/departments/aquatic->

sciences-assessment/

^11^Data from Lake Zurich were provided by the City of Zurich Water Supply (WVZ) and by the Amt für Abfall,

Wasser, Energie und Luft (AWEL) of the Canton of Zurich, Switzerland

^12^Data are from the U.S. EPA’s Long-Term Monitoring (LTM) program

**Supplementary Table 2.** Percentiles (2.5, 50 and 97.5 %) of calcium (Ca) concentrations (in mg L^-1^) and carbonate alkalinity (carbonate alk; in mg HCO_3_^-^ L^-1^) per pH category, based on long-term median concentrations for a total of 21 902 and 16 399 lake and running water sites, respectively.

| Lakes |  |  |  |  |  |  |  |  |
| --- | --- | --- | --- | --- | --- | --- | --- | --- |
| pH class | Number of sites | Ca | | |  | Carbonate alkalinity | | |
|  |  | 2.5% | 50% | 97.5% |  | 2.5% | 50% | 97.5% |
| 4.5 | 203 | 0.2 | 0.92 | 5.98 |  | 0 | 5.4 | 18.6 |
| 4.6 | 117 | 0.2 | 0.92 | 3.47 |  | 0 | 0 | 0 |
| 4.7 | 144 | 0.23 | 0.96 | 4.55 |  | 0 | 1.04 | 17.75 |
| 4.8 | 189 | 0.18 | 1.02 | 4.78 |  | 0 | 0.31 | 5.98 |
| 4.9 | 168 | 0.16 | 0.95 | 4.84 |  | 0 | 0.55 | 17.14 |
| 5 | 202 | 0.14 | 1.04 | 4.84 |  | 0 | 0.43 | 27.08 |
| 5.1 | 207 | 0.14 | 1.02 | 5.9 |  | 0 | 0.63 | 4.64 |
| 5.2 | 233 | 0.09 | 1.25 | 6.7 |  | 0 | 0.37 | 17.27 |
| 5.3 | 217 | 0.08 | 1.18 | 6.76 |  | 0 | 0.25 | 11.6 |
| 5.4 | 237 | 0.14 | 1.22 | 5.82 |  | 0 | 0.55 | 5.06 |
| 5.5 | 253 | 0.13 | 1.58 | 6.15 |  | 0 | 0.7 | 10.95 |
| 5.6 | 272 | 0.13 | 1.62 | 6.96 |  | 0 | 1.19 | 11.73 |
| 5.7 | 328 | 0.16 | 1.76 | 7.53 |  | 0 | 1.4 | 8.46 |
| 5.8 | 358 | 0.28 | 2.22 | 7 |  | 0.4 | 2.3 | 13.18 |
| 5.9 | 428 | 0.21 | 1.94 | 6.63 |  | 0.37 | 2.38 | 14.05 |
| 6 | 501 | 0.26 | 2.33 | 7.65 |  | 0.55 | 3.23 | 14.18 |
| 6.1 | 584 | 0.33 | 2.58 | 8.19 |  | 0.73 | 3.48 | 13.02 |
| 6.2 | 733 | 0.35 | 2.54 | 8.63 |  | 0.9 | 4.1 | 21.68 |
| 6.3 | 857 | 0.38 | 2.69 | 8.49 |  | 1.16 | 4.88 | 17.62 |
| 6.4 | 1059 | 0.54 | 2.97 | 9.36 |  | 1.71 | 5.55 | 20.88 |
| 6.5 | 1313 | 0.64 | 3.17 | 9.57 |  | 2.04 | 6.39 | 20.43 |
| 6.6 | 1555 | 0.76 | 3.26 | 10.2 |  | 2.65 | 7.38 | 24.35 |
| 6.7 | 1740 | 0.81 | 3.41 | 11.36 |  | 3.07 | 8.36 | 26.43 |
| 6.8 | 1813 | 1.08 | 3.71 | 12.5 |  | 3.77 | 9.15 | 34.04 |
| 6.9 | 1676 | 1.3 | 4.11 | 14.84 |  | 4.76 | 10.98 | 41.05 |
| 7 | 1399 | 1.5 | 4.63 | 17.97 |  | 6.31 | 13.23 | 51.4 |
| 7.1 | 1166 | 1.84 | 5.55 | 21.27 |  | 7.41 | 16.04 | 64.39 |
| 7.2 | 935 | 2.1 | 5.97 | 29.6 |  | 7.74 | 18.44 | 81.43 |
| 7.3 | 726 | 2.64 | 7.92 | 43.53 |  | 10.43 | 24.41 | 108.5 |
| 7.4 | 474 | 3.36 | 9.99 | 43.98 |  | 13.14 | 30.88 | 126.33 |
| 7.5 | 348 | 3.9 | 13.22 | 64.59 |  | 16.41 | 39.88 | 156.61 |
| 7.6 | 308 | 4.74 | 15.27 | 81.66 |  | 19.31 | 49.85 | 207.23 |
| 7.7 | 215 | 4.15 | 22.4 | 95.87 |  | 25.76 | 65.9 | 273.57 |
| 7.8 | 145 | 9.67 | 36.13 | 100.82 |  | 36.69 | 102.09 | 233.81 |
| 7.9 | 119 | 10 | 42.06 | 97.46 |  | 30.38 | 137.59 | 269.61 |
| 8 | 96 | 11.65 | 46.24 | 132.92 |  | 39.97 | 141.03 | 241.3 |
| 8.1 | 67 | 12.44 | 49.37 | 138.59 |  | 43.22 | 147.23 | 318.75 |
| 8.2 | 47 | 8.1 | 44.32 | 106 |  | 30.83 | 129.62 | 315.36 |
| 8.3 | 19 | 16.3 | 53.96 | 110 |  | 64.23 | 145.03 | 316.44 |
| 8.4 | 23 | 9.2 | 42.4 | 63.14 |  | 24.97 | 143.94 | 705 |
| 8.5 | 9 | 9.4 | 36 | 55.36 |  | 56.57 | 149.26 | 851 |
| 8.6 | 75 | 8.39 | 30 | 60.38 |  | 36.66 | 132.81 | 683.74 |
|  | Σlake sites  21 902 | All sites 2.5%  0.36 | All sites 50%  3.51 | All sites 97.5%  39.16 |  | All sites 2.5%  0.43 | All sites 50%  8.84 | All sites 97.5%  125.16 |

| Rivers |  |  |  |  |  |  |  |  |
| --- | --- | --- | --- | --- | --- | --- | --- | --- |
| pH class | Number of sites | Ca | | |  | Carbonate alkalinity | | |
|  |  | 2.5% | 50% | 97.5% |  | 2.5% | 50% | 97.5% |
| 4.5 | 140 | 0.05 | 1.19 | 456.92 |  | 0 | 0.62 | 18.91 |
| 4.6 | 45 | 0.35 | 1.02 | 6.96 |  | 0.61 | 1.71 | 4 |
| 4.7 | 63 | 0.15 | 1.16 | 6.04 |  | 0 | 1.8 | 6.47 |
| 4.8 | 69 | 0.15 | 1.4 | 9.4 |  | 0 | 1.16 | 11.28 |
| 4.9 | 61 | 0.26 | 1.12 | 10.33 |  | 0.24 | 1.25 | 3.75 |
| 5 | 77 | 0.17 | 1.13 | 7.4 |  | 0 | 0.63 | 14.68 |
| 5.1 | 81 | 0.5 | 1.49 | 20.93 |  | 0.06 | 1.02 | 8.46 |
| 5.2 | 75 | 0.37 | 1.46 | 6.05 |  | 0.18 | 1.53 | 5.98 |
| 5.3 | 70 | 0.63 | 1.46 | 9.21 |  | 0 | 1.24 | 6.09 |
| 5.4 | 76 | 0.34 | 1.89 | 39.95 |  | 0 | 1.28 | 26.45 |
| 5.5 | 95 | 0.58 | 1.84 | 23.33 |  | 0.2 | 1.53 | 89.05 |
| 5.6 | 101 | 0.45 | 1.96 | 9.34 |  | 0.14 | 1.83 | 10.12 |
| 5.7 | 112 | 0.67 | 1.92 | 8.84 |  | 0.36 | 2.06 | 17.7 |
| 5.8 | 133 | 0.5 | 2.05 | 9.72 |  | 0.44 | 2.75 | 34.96 |
| 5.9 | 135 | 0.5 | 2.21 | 6.98 |  | 0.72 | 2.44 | 16.33 |
| 6 | 176 | 0.51 | 2.43 | 19.15 |  | 0.75 | 3.32 | 147.57 |
| 6.1 | 184 | 0.51 | 2.47 | 17.01 |  | 1.22 | 4.08 | 25.7 |
| 6.2 | 249 | 0.94 | 3 | 21.5 |  | 1.35 | 4.96 | 39.22 |
| 6.3 | 282 | 0.79 | 3.08 | 15.52 |  | 2.21 | 5.61 | 40.22 |
| 6.4 | 415 | 1.03 | 3.2 | 15.06 |  | 2.02 | 6.05 | 33.29 |
| 6.5 | 468 | 1.14 | 3.6 | 22.14 |  | 3.32 | 7.2 | 61.85 |
| 6.6 | 577 | 1.31 | 4 | 17.5 |  | 3.69 | 8.29 | 38.2 |
| 6.7 | 626 | 1.3 | 4.4 | 25.87 |  | 3.52 | 9.8 | 61.91 |
| 6.8 | 643 | 1.5 | 5.04 | 25.52 |  | 5 | 11.95 | 60.34 |
| 6.9 | 634 | 1.39 | 5.55 | 27.05 |  | 4.6 | 12.56 | 73.92 |
| 7 | 642 | 0.91 | 5.9 | 34.17 |  | 3.36 | 15.84 | 91.85 |
| 7.1 | 593 | 1.72 | 7.3 | 52.6 |  | 6.59 | 22.56 | 154.86 |
| 7.2 | 534 | 1.59 | 8.53 | 73.9 |  | 8.29 | 26.71 | 180.04 |
| 7.3 | 563 | 1.48 | 11.05 | 69.98 |  | 7.08 | 36.45 | 192.51 |
| 7.4 | 581 | 3.01 | 13.5 | 90.81 |  | 11.52 | 44.29 | 315.1 |
| 7.5 | 559 | 3.9 | 17 | 100 |  | 10.95 | 59.39 | 275.35 |
| 7.6 | 614 | 4.2 | 21 | 132.19 |  | 16.71 | 79.11 | 330.59 |
| 7.7 | 615 | 4.15 | 27.2 | 124.12 |  | 15.39 | 87.89 | 344.86 |
| 7.8 | 759 | 4.41 | 36 | 150 |  | 16.41 | 133.75 | 341.59 |
| 7.9 | 762 | 6.77 | 49.1 | 170.96 |  | 22.16 | 166.24 | 370.2 |
| 8 | 844 | 9.4 | 52.35 | 198.75 |  | 27.94 | 177.37 | 366.69 |
| 8.1 | 960 | 10.07 | 60.98 | 185 |  | 39.34 | 200.27 | 356.23 |
| 8.2 | 738 | 11.08 | 57.35 | 168.81 |  | 45.91 | 192.63 | 391.27 |
| 8.3 | 555 | 9.88 | 55.94 | 159.58 |  | 44.97 | 198.33 | 378.21 |
| 8.4 | 300 | 12.06 | 49.5 | 146.19 |  | 58.19 | 182.22 | 390.01 |
| 8.5 | 176 | 3.93 | 44.75 | 104.48 |  | 24.46 | 157.02 | 419.61 |
| 8.6 | 267 | 10.45 | 37 | 107.6 |  | 35.92 | 141.32 | 483.94 |
|  | Σriver sites  16 399 | All sites 2.5%  0.95 | All sites 50%  11.7 | All sites 97.5%  128 |  | All sites 2.5%  1.34 | All sites 50%  42.65 | All sites 97.5%  334.85 |
